# Mechanical compression attenuates normal human bronchial epithelial wound healing

**DOI:** 10.1186/1465-9921-10-9

**Published:** 2009-02-12

**Authors:** Stephen P Arold, Nikita Malavia, Steven C George

**Affiliations:** 1 Department of Biomedical Engineering, University of California, Irvine, USA; 2 Department of Chemical Engineering and Materials Science, University of California, Irvine, USA

## Abstract

**Background:**

Airway narrowing associated with chronic asthma results in the transmission of injurious compressive forces to the bronchial epithelium and promotes the release of pro-inflammatory mediators and the denudation of the bronchial epithelium. While the individual effects of compression or denudation are well characterized, there is no data to elucidate how these cells respond to the application of mechanical compression in the presence of a compromised epithelial layer.

**Methods:**

Accordingly, differentiated normal human bronchial epithelial cells were exposed to one of four conditions: 1) unperturbed control cells, 2) single scrape wound only, 3) static compression (6 hours of 30 cmH_2_O), and 4) 6 hours of static compression after a scrape wound. Following treatment, wound closure rate was recorded, media was assayed for mediator content and the cytoskeletal network was fluorescently labeled.

**Results:**

We found that mechanical compression and scrape injury increase TGF-β2 and endothelin-1 secretion, while EGF content in the media is attenuated with both injury modes. The application of compression after a pre-existing scrape wound augmented these observations, and also decreased PGE_2 _media content. Compression stimulated depolymerization of the actin cytoskeleton and significantly attenuated wound healing. Closure rate was partially restored with the addition of exogenous PGE_2_, but not EGF.

**Conclusion:**

Our results suggest that mechanical compression reduces the capacity of the bronchial epithelium to close wounds, and is, in part, mediated by PGE_2 _and a compromised cytoskeleton.

## Introduction

Asthma is a chronic inflammatory disease marked by recurrent episodes of reversible airway narrowing resulting in dramatically increased airway resistance, work of breathing and extreme risk to the health of a patient [1]. As a result of this complex disorder, a chronic state of inflammation and wound healing exists in the lungs [2]. The persistent milieu of inflammatory mediators and growth factors are thought to contribute to structural changes in the airways including subepithelial fibrosis, smooth muscle hyperplasia and enhanced mucous secretion [3-6].

The airway epithelium serves primarily as a protective barrier between the external environment and the interior of the lung. Numerous studies have demonstrated significant denudation of the airway epithelium in the small airways in lung biopsies of severe asthmatics [7,8]. The chronic state of inflammation, a hallmark of severe asthma, appears to reduce cell viability leading to further epithelial denudation and shedding which compromises the barrier function and stimulates wound healing and abnormal and undesired tissue growth [7-10].

Additionally, the lung as a whole is a remarkably active mechanical environment which necessarily intersects with the chemical mediators of inflammation and wound healing. While mechanical strain as a result of lung expansion is thought to be the predominant force associated with pulmonary mechanics it is often overlooked that during airway constriction entire airways can close completely. Furthermore, it has been demonstrated through in vivo measurement [11], and supported by mathematical modeling [12], that asthma-induced airway narrowing results in the transmission of potentially damaging compressive forces, up to 30 cmH_2_O, directly to the airway epithelium. This phenomenon has recently garnered significant attention in the literature, and it has been demonstrated that forces of this magnitude can induce the release of inflammatory and pro-fibrotic mediators [13-15]. It has been shown in culture that the application of mechanical compression to the airway epithelium promotes collagen deposition in co-culture which may exacerbate the structural alterations in the airway wall.

While there has been significant work to characterize the response of the airway epithelium to denudation and mechanical compression independently, they are closely linked both temporally and spatially in vivo [12,16]. A single study encompassing both types of epithelial injuries and their interactions has not been reported. Accordingly, the primary aims of this study are two-fold: 1) compare the effects of two injury types, epithelial denudation (scrape wound) and airway constriction (mechanical compression), on the secretion of pro-inflammatory mediators by normal bronchial epithelial (NHBE) cells in culture; and 2) characterize how mechanical compression impacts the rate of epithelial wound healing following denudation.

We found that mechanical compression and scrape injury increase TGF-β2 and endothelin-1 (ET-1) secretion in culture, while EGF content in the media is attenuated with both injury modes. The application of compression after a pre-existing scrape wound had a synergistic effect on these observations, and also stimulated a significant depolymerization of the f-actin network. Interestingly, PGE_2 _media content declined significantly only in the setting of both injury types. Mechanical compression significantly attenuated wound healing in culture; however, this effect was partially ameliorated with the addition of exogenous PGE_2_, but not EGF. These results suggest that airway constriction may retard epithelial migration and wound healing in the airway, thus promoting a pro-fibrotic state during chronic asthma.

## Methods

### Cell Culture

Passage 3 normal human bronchial epithelial cells (NHBE) (Lonza, Walkersville, MD) were seeded at 150,000 cells/mm^2 ^on uncoated Costar Transwells^® ^inserts with 0.4 μm pore size (Fisher Scientific, Pittsburgh, PA) as previously described [17]. Following seeding, the transwells were submerged in media for 6 days to allow the cells to fully adhere and reach confluence. On day 7 the media was removed and the cells were maintained at the air-liquid interface and allowed to fully differentiate to a muco-ciliary phenotype. The media was comprised of 50% BEBM (Lonza) and 50% DMEM-F12 low glucose (Invitrogen, Carlsbad, CA) and was supplemented with growth factors provided in the SingleQuot^® ^kits (Lonza) and retinoic acid (50 nM). The experiments were replicated in two separate donors (lot numbers 4F1430 and 4F1624) of NHBE cells acquired from Lonza and were qualitatively reproducible and similar. In the interest of brevity, the data from only one donor (4F1624) is included.

### Mechanical Wounding Protocol

**Figure 1:**
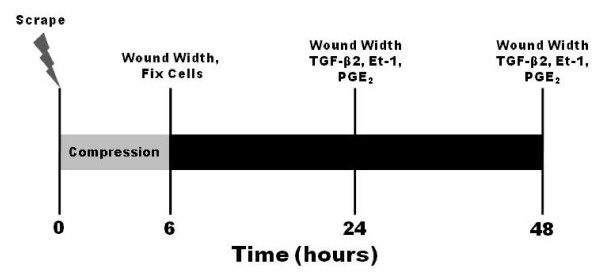
**Schematic demonstrating treatment and time course of measurements**. Cells slated to undergo scrape and compression were scraped with a 200 μl pipette tip at time zero and washed 2× with cell culture media to remove debris and were imaged. The wells then underwent 30 cmH_2_O mechanical compression for 6 hours. Following compression the cells were imaged and a representative well was fixed for imaging. 24 and 48 hours following the initial insult cells were again imaged to determine wound width, a representative well was fixed for imaging and media samples were taken for mediator content.

Following differentiation, cells were randomized into one of four groups (n = 6): 1) untreated control cells, 2) scrape wound only, 3) scrape and compression wounds, and 4) compression wound only. Media was changed 16 hours prior to the beginning of the experiment, and at time zero cells were exposed to their respective injury. A schematic of the experimental protocol is shown in Fig. 1. Briefly, cells that were slated for scrape wound only and scrape and compression wounding experienced a single scrape with a 200 μl pipet tip, lengthwise across the diameter of the Transwell, resulting in a wound width of approximately 500 μm. The wells were then washed 2 times with 50:50 media to remove debris and non-adherent cells and were imaged with a 4× objective. Cells undergoing scrape wound only were returned to the incubator, while cells slated for scrape and compression were exposed to 30 cmH_2_O compression for 6 hours (see description below). A six hour exposure to compression is similar to the range (1–8 hours) from previous studies [13,18], and is similar in magnitude to the duration of an acute asthmatic episode. Cells destined for compression wound only were simply exposed to the 6 hours of mechanical compression 16 hours following the media change and then returned to the incubator. At 6 hours wells that underwent a scrape wound were again imaged. At 24 hours all cells were imaged again and media was collected from 5 wells for analysis with some wells fixed for immunofluorescent imaging (see below). Finally, at 48 hours the remaining 5 wells were imaged, fixed and the media was collected. All media was stored at -20°C until ELISA analysis for active TGF-β2, ET-1, PGE_2 _or EGF (R&D Systems, Minneapolis, MN). Three additional groups of cells were exposed to mechanical compression with pretreatment with 900 pg/ml PGE_2_, 150 pg/ml additional EGF or both, and imaged at the respective time points.

### Mechanical Compression Device

The method for applying mechanical compression to cells in culture is based on systems described elsewhere [18]. Briefly, a simple aquarium pump provides airflow to a flow controller set to approximately 3 ml/minute. Proximal to the flow controller, the tubing is connected to any number of stoppers which fit tightly into a Transwell insert containing cultured NHBE cells where the compression is applied. Each stopper also contains an outlet port allowing airflow out of the insert, which leads to an external water trap. System pressure is controlled directly by the depth of the outlet port in the water trap. The entire system, with the exception of the water trap, is contained in a controlled incubator environment at 37°C, 5% CO_2 _and 100% humidity.

### Scrape Imaging

**Figure 2:**
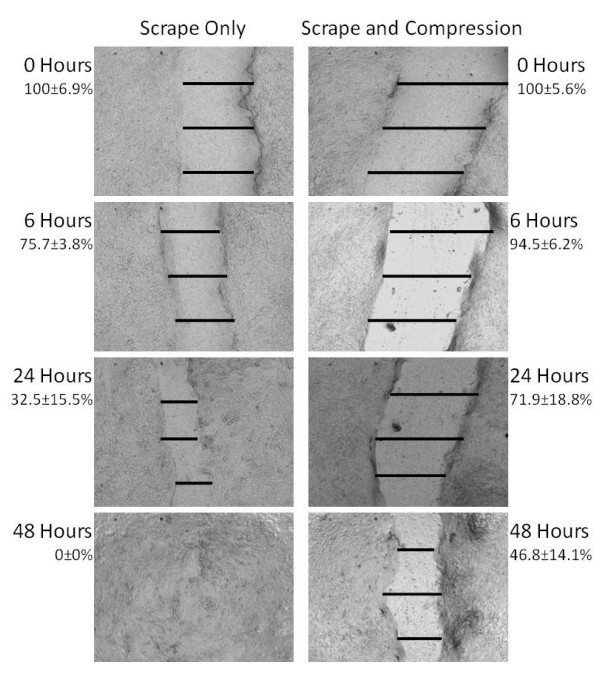
**Representative images of wells that underwent scrape only (left) and scrape with 6 hours of mechanical compression (right) at 6, 24, and 48 hour time points**. The black bars in each image represent three wound width measurements that were subsequently averaged, normalized to the initial wound width and treated as a single measurement (text).

Scrape wounds were imaged with a 4× objective using an Olympus 1X51 microscope (Olympus America, Center Valley, PA) and a QImaging QICAM 12 bit camera with Qcapture Pro acquisition software. Each well was imaged along the entire length of the scrape wound. From each image, three representative widths were measured from the top, middle and lower third of the scrape and averaged using Image J software (NIH Image J, Bethesda, MD). Examples of measurements are shown in Fig. 2. Wound healing for each time point was then normalized with respect to original wound width.

### Immunofluorescence Microscopy

At assigned time points, cells slated for f-actin labeling were fixed in 4% formaldehyde for 20 minutes, and those slated for the labeling of microtubules were fixed in -20°C methanol for 2 minutes. Both groups were subsequently permeabilized with 0.5% Triton X-100 for 10 minutes, and nonspecific binding was blocked by incubation in 10% goat serum for one hour. Cells were rinsed with PBS and incubated in either 5 μg of Alexafluor 568 phalloidin (Invititrogen, Carlsbad, CA) in 200 μl PBS or monocolonal anti-a-tubulin (Sigma, St Louis, MO) 1:250 dilution in PBS for two hours followed by an overnight incubation in Alexafluor 488 anti-mouse secondary antibody (Invitrogen) diluted in PBS 1:500. Following staining, cells were rinsed with 0.1% Triton X-100 four times and the Transwell membranes were carefully removed with a scalpel and mounted on a microscope slide. Cells were then imaged with a 20× or 40× objective.

### Statistical Analysis

All data is presented as mean ± standard error. Each image was treated as a separate sample and all data were analyzed with SigmaStat software package (Jandel Scientific, San Rafael, CA) by one way ANOVA, bonferroni test. Differences between groups were considered statistically significant for p < 0.05.

## Results

Two examples of the progression of wound healing for cells that underwent a scrape wound (left) and those that experienced both scrape and 6 hours of mechanical compression (right) are shown in Fig. 2. In this case, the images for each treatment are located at approximately the same site along the scrape wound. Each bar represents one measurement of the wound width and the average width of the three measurements normalized with respect to the original width is represented next to the image and considered a single measurement. It can be seen that the non-compressed wound closes completely within forty eight hours, while the compressed wound only closes slightly more than half of the original scrape distance.

**Figure 3:**
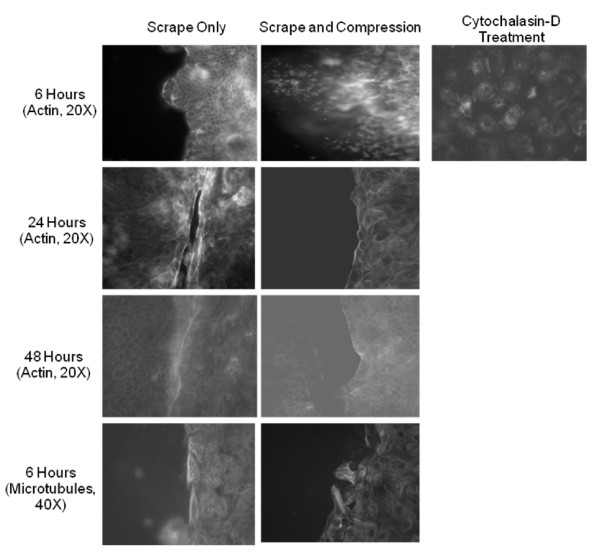
**Representative images of filamentous actin staining of fixed cells at 6, 24 and 48 hour time points that underwent a scrape wound (left), scrape and compression wound (middle), or cytochalasin-D treatment (right)**. Also included are images of microtubule structure (bottom row) at 6 hours.

The effects of scrape (left) and mechanical compression and scrape (right) on the cytoskeletal components, actin network (20×) and microtubule network (40×), can be seen in Fig. 3. At 6 hours in the scrape only condition, the actin network is a rich network of primarily cortical actin that extends throughout the epithelium. This is in stark contrast to the appearance of the actin network in cells that underwent mechanical compression and are lining the wound, in which the network is completely depolymerized with no discernable network. These observations are qualitatively very similar to the panel in the 3^rd ^column showing a group of cells that underwent cytochalasin-D treatment to chemically depolymerize the f-actin network. By 24 hours partial recovery in the f-actin network is evident, and essentially complete by 48 hours at which time the wound is nearly closed. Surprisingly, the microtubule network demonstrated no significant reorganization as a result of mechanical compression across all time points, (6-hour time point, Fig. 3, bottom panels). It should be noted that cells underwent viability assays for each treatment at every time point, and there were no discernable differences between groups (data not shown).

**Figure 4:**
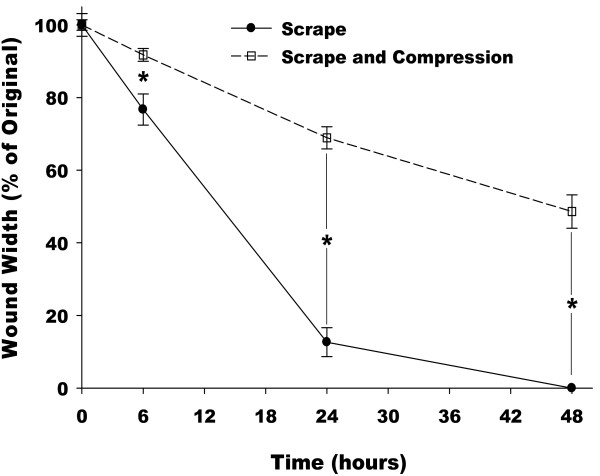
**Wound width normalized with respect to initial wound width as a function of time for cells that were scraped (circle, solid line) or scraped and underwent mechanical compression (square, dashed line)**. (* p < 0.05 between groups).

The population averages for wound closure are presented in Fig. 4. The cells exposed to the scrape wound (solid line) close to approximately 10% of the original width after only 24 hours, and all wounds in all images were completely closed by 48 hours. In contrast, the cells that were also compressed closed to approximately 50% of the original wound width by 48 hours. These differences are statistically significant at all time points following the wound and compression. It should be noted that in both treatments wound closure appears to progress in a linear manner, and with the average wound width of the scrape only cells reaching zero at approximately 28 hours.

**Figure 5:**
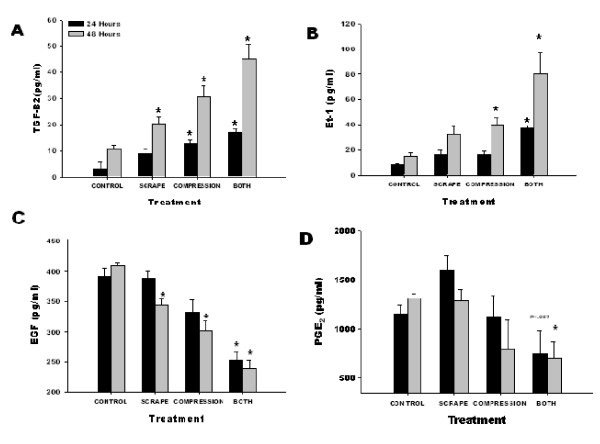
**(A) Active TGF-β2, (B), Et-1, (C) EGF, and (D) PGE_2 _media content for unstimulated cells (control), cells that underwent a scrape wound (scrape), cells that underwent mechanical compression (compression), and cells that underwent both a scrape wound and mechanical compression (both) at 24 (Black) and 48 hours (Gray)**. (* p < 0.05 compared to control at respective time point).

Active TGF-β2, ET-1, EGF and PGE_2 _media content at 24 and 48 hours for the scrape only, compression only, and scrape and compression treated cells are shown in Fig. 5. Active TGF-β2 and ET-1 (Fig 5A and 5B) demonstrated similar trends whereby their content increased as a result of both scrape and compression wounding and, in general, showed an enhanced response as a result of compression relative to scrape wound. At 48 hours there was a significant increase in active TGF-β2 content for both scrape only and compression only injuries whereas ET-1 content only reached significance as a result of mechanical compression at that time. When cells were exposed to both compression and scrape wounding there was a significant increase in magnitude of secretion at both time points in the case of both active TGF-β2 and ET-1.

The results for media analysis for EGF (Fig 5C) appear to be slightly more confounding. It should first be noted that the media is supplemented with EGF at 550 pg/ml in order to promote proper proliferation and differentiation of the NHBE cells at an air-liquid interface [17,19]. With this in mind, 48 hours following a scrape wound or compression induced injury EGF media content decreased significantly, in comparison to control cultures, with a slightly higher decrease in response to compression. The combination of injury types produced a more profound response with media content decreasing significantly at both 24 and 48 hours to approximately 250 pg/ml (150 pg/ml less than control cells), which is less than half of the original media content.

PGE_2 _media content is shown in Fig. 5D. Relative to control, there was no significant differences between PGE_2 _content as a result of compression or scrape alone; however, in combination, there was a decrease in content at 24 hours that approached significance (p = 0.057) and a significant decline at 48 hours. It should be noted that at both time points these declines were also significant in comparison to scrape only wounds.

**Figure 6:**
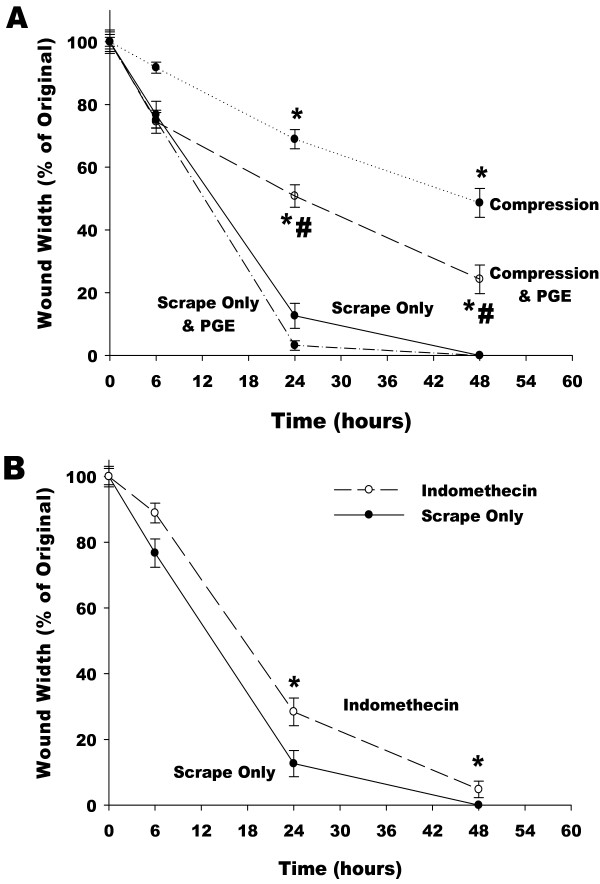
**Wound width normalized with respect to initial wound width as a function of time (A) for cells that were scraped (circle, solid line), scraped and underwent mechanical compression (circle, dotted line), scraped, underwent compression and treated with PGE_2 _(circle, dashed line) or scraped and treated with PGE_2 _(circle, dash-dot)**. Wound width normalized with respect to initial wound width as a function of time (B) for cells that were scraped (circle, solid line or treated with Indomethacin (circle, dashed line). (* p < 0.05 compared to scrape only, # p < 0.05 compared to scrape and compression).

In order to directly test if the decline in media content of EGF or PGE_2 _was responsible for the attenuation in wound healing in the presence of mechanical compression the experiment was repeated in the presence of exogenously added PGE_2 _(900 pg/ml) or EGF (150 pg/ml) to offset the effects of mechanical compression on mediator content in the media. The results are shown in Fig. 6A. EGF supplementation had no effect on the rate of wound healing (data not shown); however PGE_2 _supplementation resulted in a significant improvement in the rate of wound healing at 24 and 48 hours in the compressed cells, and a slight, however insignificant, improvement in scraped cells not exposed to compression. Additionally, treatment with Indomethacin, a Cox-1 and -2 inhibitor that suppresses PGE_2 _synthesis, resulted in a significant reduction in the rate of wound healing at all time points following the scrape injury (Fig 6C).

## Discussion

The primary objective of this study was to directly test in cell culture the effects of mechanical compression on the rate of epithelial wound healing following denudation. Both conditions are present simultaneously in the setting of diseases such as chronic asthma. Although utilization of a scrape wound or mechanical compression are utilized as means to simulate epithelial injury thought to arise in vivo during chronic asthma, a direct comparison of these injury models and the interdependence has yet to be presented. The principal findings of this study are three-fold: 1) mechanical compression significantly attenuates epithelial migration and wound healing in NHBE cells in culture; 2) the underlying mechanism is, in part, PGE_2_-mediated; and, 3) mechanical compression results in significant depolymerization of the actin network in these cells. We further suggest that this degradation of the actin cytoskeleton also exerts a negative influence on cell motility and wound healing. Mechanical compression and scrape wound have a similar influence on inflammatory mediator release, and the simultaneous application of these injuries has an even more exaggerated effect. However, it is obvious that mechanical compression has a dramatic effect on actin structure not seen with scrape injury. This observation may influence cell behavior beyond wound healing not considered in this investigation.

EGF and PGE_2 _have been shown to have a significant role in lung epithelial wound healing [20,21], while TGF-β2 and ET-1 are more influential as pro-fibrotic mediators [13,22]. Interestingly, we observed an increase in the media content of both pro-fibrotic mediators, while observing a decline in the mediators related to wound healing. This would suggest that mechanical compression in the setting of epithelial denudation may not only attenuate the ability of the airway epithelium to repair itself, but further exacerbate airway remodeling and fibrosis during chronic asthma.

Tissue culture techniques have been utilized to characterize the effects of epithelial denudation on the inflammatory response and wound healing of the airway epithelium. Thompson et al. [22] co-cultured normal human bronchial epithelial (NHBE) cells with pulmonary fibroblasts and found that a scrape wound enhanced TGF-β2 secretion, and resulted in a subsequent pro-fibrotic response of pulmonary fibroblasts. Puddicombe and colleagues [23] also reported an increase in TGF-β2 secretion after scrape in bronchial epithelial cells, and found that epidermal growth factor receptor (EGFR) appeared to play a key role in wound healing [24]. Savla et al. [25,26] addressed the relationship between mechanical forces and wound healing when they demonstrated that mechanical strain (not compression) inhibits repair of the airway epithelium in cells grown on a silastic membrane. This group has also shown that cyclic stretch inhibits prostanoid synthesis, including PGE_2_, in the airway epithelium [27], and they later demonstrated PGE_2 _is a key factor in regulating wound closure in the airway epithelium [20], although they did not specifically link decreased PGE_2 _synthesis with inhibition of epithelial repair as a result of strain. While this group utilized strain rather than mechanical compression, there are parallels with our current study. We did not examine PGE_2 _synthesis specifically, but we did demonstrate that PGE_2 _media content is significantly decreased as a consequence of mechanical compression. In light of these previous reports, we believe that this observation is a result of reduced secretion of PGE_2 _into the extracellular space, as opposed to increased consumption. This is supported by our observation, that replenishing PGE_2 _in the media partially recovered the rate of wound healing.

An interesting observation is that EGF media content decreased as a result of a scrape wound, mechanical compression, and even more so when both injury types were applied. Puddicombe and colleagues [23] have demonstrated that mechanical compression upregulates the expression of EGFR. Accordingly, our data demonstrating increased EGF consumption is consistent with enhanced EGFR upregulation ultimately leading to increased EGF binding, internalization, and consumption. This further suggests that the decrease in media EGF content following the mechanical insults was not due to decreased autocrine EGF synthesis and/or secretion, but a consequence of increased consumption. Given the large body of evidence demonstrating the significant role of EGFR activation in wound healing, our observations that additional exogenous EGF did not impact the rate of wound healing suggests that the EGFR s are either saturated from pre-existing EGF content in the media, or EGFR regulation in the form of synthesis and/or trafficking of the receptor to the cell membrane is a primary mechanism of EGF-directed wound healing.

In order to achieve a properly differentiated phenotype of airway cells reflective of what is observed in vivo (ciliated columnar, mucous producing cells with tight junction formation), the NHBE cells must be grown at an air liquid interface with specialized media. One necessary growth factor to obtain proper cellular characteristics is EGF, present in properly formulated media at approximately 550 pg/ml. It has been widely demonstrated that EGF expression is upregulated as a result of both mechanical compression [14] as well as a scrape wound [28] in a number of cell types, and is a central factor in epithelial wound healing. While it is not clear what baseline level of EGF would correspond to the in vivo condition, it would appear that EGF consumption outpaces secretion suggesting that EGF levels present in the media are likely elevated with respect to what one would expect at equilibrium. In effect, this would likely result in a saturation of EGF receptors (EGFR) and eliminate the EGF mediated regulation of wound closure in our model.

Cytoskeletal remodeling is a vital process in the progression of wound healing. Accordingly, we examined the effects of mechanical compression on the two major cytoskeletal structural protein networks, and more specifically, the relation of these structures to wound healing. We observed a nearly complete depolymerization of the f-actin network following 6 hours of mechanical compression which should significantly reduce cell motility and wound healing. We utilized an additional Transwell of differentiated NHBE cells and applied a scrape wound in the presence of 10 μg/ml of cytochaliasin-D. Not surprisingly, wound healing was nearly completely retarded in this case (data not shown).

The cells that were injured with only a scrape wound appeared to heal in a manner well documented in other studies of wound healing in epithelial cells. In these cases a pronounced band of actin is present at the wound edge. This purse string formation was documented by Lotz et al. [29] and is also apparent in our scrape only images; however, the formation of the structure is noticeably absent in the early image of cells that also underwent mechanical compression, likely serving to inhibit the progression of wound healing. Lotz et al. [29] also documented the protrusion of lamellar bodies into the wound space in injured intestinal epithelial cells. In our images these structures were not immediately obvious in either the compressed or non-compressed images. While it may be possible that these structures were absent, it is more probable that increased background fluorescence as a result of the Transwell membranes made viewing these structures not possible.

Another key process present during *in-vivo *wound healing and other tissue culture models of wound healing that was not directly examined in this study is cell proliferation. Fully differentiated confluent NHBE do not proliferate significantly in culture once confluent, and wound closure following a scrape injury has been shown to be independent of cell proliferation [30]. Accordingly, the influence of compressive forces on cell proliferation and the rate of wound closure in not likely to be a significant factor in the current experiments.

Here we present the novel fining that mechanical compression results in significant f-actin depolymerization however the mechanism is not abundantly clear. One possibility is that mechanotransduction of these forces results in an intercellular signaling cascade that ultimately leads to cell directed depolymerization of the network. If this is the case, the exact nature of this cascade is beyond the scope of this study; however the biological advantage to this type of response is not obvious. A more likely option is that the physical force imposed by compression physically altered the structure of the network or interfered with the dynamic nature of the actin networks preventing f-actin polymerization from g-actin monomers. A link between PGE_2 _regulation and maintenance of the actin network is a distinct possibility; however the limited evidence in the literature suggests that actin depolymerization results in an upregulation of PGE_2 _synthesis [31]. This would be contrary to our findings and suggests that our observations are not intrinsically linked. Regardless, mechanical force induced reorganization of the actin network, and the determination of the link with PGE_2 _regulation, if any, certainly merits further investigation, and it is quite likely there are other factors involved not specifically addressed in this investigation.

In conclusion, we have demonstrated that mechanical compression attenuates epithelial wound healing under conditions thought to arise as a result of airway constriction during chronic asthma. These observations are, in part, PGE_2_-mediated and it is highly likely that mechanical compression induced f-actin depolymerization also interferes with wound healing in this model. Our findings suggest that airway constriction further aggravates the pre-existing, pro-inflammatory state of the denuded airway epithelium. The accompanied increase in inflammatory mediator release in conjunction with a compromised barrier function of the airway epithelium likely serves to accelerate the progression of airway remodeling and the deleterious consequences on lung function during chronic asthma.

## Competing interests

The authors declare that they have no competing interests.

## Authors' contributions

SA designed, planned, and performed all of the experiments, and wrote significant sections of the manuscript; NKM assisted in SAEC culture and data interpretation; SCG provided overall guidance for the study, assisted in the experimental design, analysis and interpretation of the data, and writing of the manuscript. All authors have read and approved the final manuscript.
